# Under one roof: Strategic intersectionality among women negotiating the Calais border under lockdown

**DOI:** 10.1177/23996544231173546

**Published:** 2023-05-16

**Authors:** Maria Hagan

**Affiliations:** Department of Social Anthropology, University of Manchester, Manchester, UK

**Keywords:** Calais, migrant women, COVID-19 lockdown, domestic space, strategic intersectionality

## Abstract

Calais has attracted the attention of numerous scholars since it emerged as a key European migration pressure point in the early 1990s. Yet in-depth discussions relating to the experiences of displaced women at this border remain rare. This article draws on my unexpected experience of spending 3 months in lockdown with border-crossing women in Calais when the field research I had been carrying out with (predominantly male) people living in makeshift camps at the border was interrupted by the COVID-19 pandemic in March 2020. Drawing on the work of feminist geographers I conceptualise the northern French border as a *virilised* space, where policing that imposes harsh living conditions at the border reinforces male subjectivities and exacerbates gender-based exclusion and violence. Drawing on ethnographic insights from this intimate period of living together, I then detail how lockdown prompted the women I was living with to renegotiate this terrain with physical proximity to their male counterparts ruled out. I argue that the role of domestic space changed during this period, from one of hindrance to the mobility of the female body to one of strategic potential. In the light of these findings, I propose a conceptualisation of the lockdown period as a moment of retreat and rupture that facilitated these women’s engagement in *strategic intersectionality*, drawing on their unique positions as a small but diverse group to endure crisis and negotiate opportunities to reach the United Kingdom.

*I watch as Haben* ^1^ *takes a few bank notes from a hiding place at the foot of her bed. Using the windowsill as a surface to lean on, she flattens the notes with the palm of her hand, then starts to roll them up tight. It’s dark outside, and she pauses in her task to pull a crack in the curtains shut. Senait sits in front of her, ready, on a stool. She lets her hair down and it falls in long, thin braids down her back. Cash-roll in one hand, Haben takes Senait’s hair in the other and starts to weave a few braids around it, tying the money as tight as she can to the crown of her head. When satisfied it holds, she ties the braids together in a large, high bun so the money is well out of sight. ‘To keep it safe,’ Senait grins, catching my eye as she pulls a grey winter hat tight over her hair. Now I understand the hours her friends spent weaving in her extensions yesterday. ‘During the war with Ethiopia, our mothers in Eritrea would carry messages from one city to the next in this way,’ Haben explains. Senait moves towards the dresser and grabs a used plastic bottle of Lipton Ice Tea, half full of holy water blessed months ago by the young deacon who leads Sunday mass on the parking area by the men’s encampments. Despite the warmth of the room, the women pull on layers of clothing, ready for the midnight cold off the Channel. Again, Haben taps open the Windfinder weather app on her phone, trying to reassure her friend who, jubilant just minutes ago, has suddenly fallen sombre and silent: ‘The waves are small tonight Senait, it is good.’ An hour later, the call finally comes and they slip out into the night. The following afternoon, their excited voices inform me of their safe passage; a stream of nervous relief through a stranger’s mobile phone. Soon after, a BBC News report documents yet another abstract ‘small boat of women and men landed on the beach at Folkestone.’*

Field diary, Calais 15.05.20

## Introduction

I was less than halfway through six months of ethnographic research in Calais when the French state declared a national lockdown in mid-March 2020, in response to the rapid spread of COVID-19. I had recently started living-in at a safehouse for vulnerable people near the border city, and when the lockdown was announced went into strict confinement with the house residents: women from Ethiopia, Iran, Eritrea, Sudan and Kurdistan, living at the northern French border while either seeking asylum in France or to cross the Channel to the United Kingdom. We lived together under the exceptional measures the French government introduced, imposing that people stay inside their homes, limiting their outings to a bare minimum and requiring people carry a signed document justifying their presence in public space (Legifrance 2020). This article presents the findings of the feminist ethnography I inadvertently came to carry out in these conditions, ‘drawing on methodological strategies that embrace the everyday experiences of people, especially those forced to live on the margins, as epistemologically valid’ (Davis 2013: 27). The unexpected and intimate experience of living together in crisis for 3 months lent depth to my understanding of the lives and experiences of border-crossing women in Calais.

It is no secret that since the early 1990s, displaced people have been drawn to northern France in pursuit of clandestine passage to the United Kingdom. Less acknowledged has been the consistent presence of women alongside men at this border, albeit in much smaller numbers. Although sophisticated discussions have explored the political implications of the spectacular makeshift camp known as the Calais Jungle that existed at the border from 2015 to 2016 (Agier et al. 2018), have scrutinised its materialities (Mould 2018; Hicks and Mallet 2019; Katz 2017) and the racial politics of its emergence and demolition (Davies and Isakjee 2019; Davies et al. 2017), discussions of women’s experiences have remained tangential, often appended to those of men or reduced to nondescript categories like ‘vulnerable’ or ‘women and children,’ glossing over gender-specific aspects of their border experience. This article joins a growing body of literature that challenges the protracted absence of interest in the experiences of women who migrate (Tyszler 2018; Sahraoui 2020; Schmoll 2020; Soudant-Depelchin 2016; Pickering and Cochrane 2013; Lutz 2010). Since the early 2000s, feminist scholars have been rethinking how power, place and experience intersect, reconceptualising geopolitics as embodied, intimate and fundamentally bound up with the materialities, practices and visceral experiences of everyday life (Torres 2018; Massaro and Williams 2013; Hyndman 2001, 2004; Dowler and Sharp 2001). Taken broadly, this approach challenges top-down conceptualisations of power and geopolitics, while pushing for an intersectional, critical approach that draws attention to the effects of geopolitics on a variety of often-neglected subjectivities. In this vein, this article interrogates migrant women’s absence from the literature on Calais while providing insight to the everyday fears, violence and dilemmas they face at this border, as revealed through the intimate experience of living together under lockdown.

This article first fleshes out the context for this analysis, conceptualising northern French border space as profoundly *virilised* (Tyszler 2018). Tyszler uses this valuable concept (drawn from her ethnographic work at the Morocco-Spain border) to express how border securitisation strategies that enforce a harsh environment reinforce male subjectivities, in turn exacerbating gender-based violence against women and their exclusion (ibid; 2019). I then elaborate on the theoretical underpinnings of this article, drawing on the work of feminist geographers to discuss the gendered implications of border virilisation, and to unpack how differently positioned women united by a common goal might engage in *strategic intersectionality* (Ramirez et al., 2006). After outlining the methodological approaches and ethics involved in this research, I elaborate on the empirical material underpinning this article’s argument. I argue that the changed spatial conditions brought about by the lockdown momentarily disconnected the women I was living with from the virilised border environment and the gendered social structures upon which they were dependent. The lockdown recast domestic space as advantageous, prompting the women to build strategic intersectional relations with one another and create new border-crossing opportunities for themselves. I refer to the women at the heart of this article as female border-crossers, preferring this active referent to the nondescript and passive ‘migrant women.’

## Being a woman at the calais border

‘Calais is not a good place for a woman.’

Asmerom, Eritrean male youth, Calais 05.02.20

Far fewer women than men attempt to make the crossing from France to the United Kingdom via Calais: in February 2016, fewer than 4% of Calais ‘Jungle’ residents were women and girls (*Help Refugees* census cited in Hangal, Paton et al., 2016). The sprawling Jungle camp was demolished in October 2016, and ever since the French government has sought to toughen its border by exposing migrant people to the elements through a ‘zero camp tolerance’ policy: a hostile environment is inflicted through the routine, systematic raiding or eviction of makeshift encampments (Hagan 2022). This profoundly racialised violence seeks to dehumanise border-crossers of all genders, who in this context resort to living furtively in forests and industrial zones at the border (ibid; Palmas 2021). Evictions render women particularly vulnerable by depriving them of even the flimsiest threshold beyond which to take refuge. In other words, without directly attacking women, the French authorities exacerbate the fear and threat of gender-based violence they experience, encapsulating how border securitisation contributes to exposing women to violence and thus to *virilising* border space (Tyszler 2018). In these conditions, numbers of women passing through the border zone dropped: only 50 women are thought to have passed through Calais in 2017 (Richards and Timberlake 2019) and just 35 between November 2018 and 2019, most of whom were Iranian and travelling as part of a family, or Eritrean and Ethiopian women travelling alone (ibid). When I asked Ariana, a young Iranian woman, if she knew why there were so few women in Calais in early 2020, she replied:

‘Less of us leave our country. That’s the point. Because even at the first step, the men were *too many more* than the women… It’s not very usual for women to leave their home in this way. It’s very difficult. It’s the *man’s* way. But that’s a good question, where are the women? You know, many men I met along the way told me I would never make it alone. […] We who arrive here, we are the survivors.'

This notion that women in Calais are ‘survivors’ of a male-dominated trajectory emphasises the importance of taking a feminist approach to the study of virilised terrain (Tyszler 2018). Indeed, the northern French border zone is a highly gendered geography; a ‘man-made environment’ (Pain 1991) emergent at the intersection of the dispossessive practices of the French authorities and the male peers in migration with whom women interact. After the demolition of the Jungle, in the context of routine police raids on encampments, *Gynécologie sans Frontières* (GSF) began to operate ‘a daily, 12-hour search for vulnerable girls through forests, swamps and industrial parks’ (Rowsome 2018), painting a fearsome image of the living conditions women face:

‘Women are more exposed to sexual violence when eviction operations take place, as GSF teams observed through the recrudescence of violence against women. Testimonies of sexual violence increased as well as requests for abortions. Despite all of the criticisms we may have about the jungles^2^ and camps in terms of delinquency with smugglers, promiscuity and the risk of sexist violence; at least they are identifiable places regulated by a certain codification, a social tissue and in which we can intervene more rapidly to assist victims of violence against women’ (author’s translation, GSF 2018: 13).

This emphasises how a state strategy that targets shelter has devastating gendered implications, exposing women to the heightened risk of violence generated when people are dispersed from places where protective social structures and familiar environments exist (Valentine 1989). During evictions, social structures that might protect women are splintered: people are scattered and incentivised to conceal themselves, reducing women’s visibility not only to the authorities, but also to their peers and the associations that support them. This emphasises the profound intertwinement of geopolitics with the intimacies of everyday life (Pain and Staeheli 2014): in Calais, displaced women carry a vivid sense of insecurity with them, exacerbated by the fear that comes with navigating tense spaces in which male peers, who outnumber them, are also worn down, often on edge and exhausted. Nazyul, a young Kurdish woman living in a northern French encampment with her husband up until she went into labour, described the fear and insecurity she felt living there as a pregnant woman: ‘I couldn’t hold my urine well anymore near the end of my pregnancy, but going out of the tent at night is dangerous and you see horrible things…’ Overlooked by her peers and by the authorities as a minority if not an anomaly, the border-crossing woman becomes marginal, collateral. In the words of bell hooks: ‘to be in the margin is to be part of the whole but outside the main body’ (2015 [1984]: xvii). Routine violence targeting women, and especially women of colour (Crenshaw 1991), is compressed and intensified in the microcosm of the border.

With an estimate of just 25 border-crossing women in Calais when the first French lockdown was announced, shelter was available for most. However, lockdown meant that these women faced the dilemma of either confining themselves to shelter, or living full time in the encampments alongside the men and in deplorable conditions. The virilisation of the border was further intensified during this time: throughout the lockdown and despite the threat of infection, the authorities refused to cease evictions. Displaced people and human rights groups reported increased instances of police violence, while people’s access to humanitarian support was heavily compromised by sanitary state of emergency regulations and intensive policing of non-state humanitarian action (Hagan 2023).

## Strategic intersectionality in a virilised border zone

Feminist geographers have long observed how the gendering of space reinforces power and difference, as reflected and reinforced in the spatial arrangement of a given place (Rose 2021; Pain 1991; Bowlby et al., 1982; McDowell 1999). Migration as a phenomenon has taken on profoundly gendered connotations: those who stay put in one place (namely a refugee camp) for a protracted period, waiting indefinitely with an unresolved administrative status, are feminized (regardless of gender), whereas those who travel onwards to the global North are framed in masculine terms and as threats to security and the welfare state (Hyndman and Giles 2011: 363). Illustratively, while the important role of women as family providers in refugee camps has been discussed in literature on displacement (Freedman 2016: 579), there has been little discussion of women travelling alone or with other women and providing for themselves and their female peers. This leaves women in migration discursively trapped within certain tropes incompatible with the figure of the solo, female border-crosser - a tendency this article begins to reverse.

Black feminists emphasise how space is often rendered antihuman for women, and namely for women of colour (hooks 1995; McKittrick 2006). The previous section illustrated how the Calais border zone is a racialised and virilised geography *designed* as unliveable for all migrant people, and especially for women. As Hedge writes: ‘the enactments of power and policing at the border amplify and solidify existing systems of race and sexual discrimination’ (2021: 1670). Spatial manipulations compromise the migrant woman’s ontological survival, emphasising the ‘spatial unrepresentability of black femininity’ (McKittrick 2006: xxv). Women are cast as ‘space invaders’ (Massey 1994; Puwar 2004), not only of French national space, but also of border space dominated by their male peers in migration (Tyszler 2019, 2021). They are constantly reminded of their place through the violent ‘intimacy-geopolitics’ (Pain and Staeheli 2014) of these spaces, through practices of gendered, ‘everyday bordering’ (Yuval-Davis et al., 2018). In such contexts, the solo female border-crosser embodies she who Ahmed (2017) conceptualises as the ‘wilful woman,’ a social frame that defines women in terms of their disobedience of the norms that society seeks to impose. The wilful woman must fight and persist in acts considered disobedient to exist, which in some cases may ‘legitimise’ violence against her (ibid). While migrant people as a whole are punished at the border by the state for engaging in what it perceives as an ‘unruly’ mobility (Tazzioli 2020), wilful migrant women are doubly punished (be it actively or passively) for their transgression, by the state but also by male peers on social grounds, through their exclusion from certain spaces and opportunities for passage. As Rose writes: ‘women are always guilty, either of having too much human agency, or not enough’ (2021: 340). Indeed, women at this border *must* forge a place for themselves to be in with a chance of crossing the border: ‘the reconfiguration of space becomes a question of ontological survival and the future’ (Gökarıksel et al., 2021: 11).

Gökarıksel et al. question how we may dismantle or live apart from structures of race and gender written into the built environment (ibid). This article argues that the changed spatial conditions entailed by the pandemic-prompted lockdown emerged as an unexpected moment for women to step away from, if not dismantle, certain aspects of the virilised border environment and social structures upon which they were formerly dependent. Women living in shelter found themselves physically severed from the space of the encampments, and were pressed to reconfigure the space of the shared home and the social ties it offered as opportunities. As Ho and Maddrell observe, the pandemic created new experiences of vulnerability which ‘reconfigure individual and collective emotional-affective landscapes’ (2021: 3), enabling new practices and social ties to emerge. This article rethinks the role of the safehouse at a time of lockdown, commonly considered a space of restriction and limitation, as a reconfigured space of opportunity.

The subjectivities of female border-crossers are of course varied, and this article aims to show how these women mobilised their differences strategically in the reconfigured context of lockdown. Crenshaw’s landmark work on intersectionality (1991) emphasises how gender subjectivities are not homogenous but diverse and multiple, shaped by a multitude of intersecting social and political characteristics such as faith, class, sexuality, age and so on. Intersectionality is an antiracist and feminist tool which, when acknowledged, may enable us to unravel systems of power and discrimination (ibid; Yacob-Haliso 2016); transformative change may emerge from acknowledging strength in non-dominant difference (Lorde 1984). Ramirez et al. draw on this work to develop the notion of *strategic intersectionality* in reference to female Latina legislators in policy-making circles in the United States, arguing that these women may draw upon their intersectional positions to their strategic *advantage* within policy-making processes: ‘the intersection of gender and ethnicity might position Latina legislators to have a richer set of strategic options’ (Ramirez et al., 2006: 7). This article brings this concept to the private space of the safehouse and reworks it: rather than focusing on the strategic opportunities presented by *one* woman’s varying positionalities, it takes the combination of positionalities of a *group* of differently situated women, working in coalition to engage in *strategic intersectional* acts. Similarly, Carastathis argues for a revival of readings of intersectionality as creating ‘unity across lines of difference’ rather than narrowing down concepts of shared identity (2013, 942; see also Cole 2008). This amounts to weaving alliances across difference and mobilising that difference as an asset. These coalitions emerge out of *necessity* (Carastathis 2013); rooted in common crisis and these women’s recognition of one another as wilful actors with a common goal of border passage. Bearing in mind that not all women in Calais would self-identify as feminist or consider themselves engaged in a gender-based political act by migrating, Ahmed’s (2017) concept of the wilful woman befits the border-crossing woman who persists on a dangerous migration journey despite social convention, strategizing with female peers to find a way to cross. This begins to capture how border-crossing women came to strategically reappropriate domestic and social space under lockdown, stretching the parameters of their own opportunities as a heterogeneous group to make the border zone a ‘more humanly workable’ space (McKittrick 2006: xii). The empirical discussion that follows examines how this played out at the intimate scale of the home.

## Methodology: An unexpected, intimate cohabitation

Tyszler points out that journalists (like researchers) tend to justify their lack of investigation of women’s conditions at the border with the arguments that women are ‘a minority’ and ‘difficult to access’ (2018: 83). These excuses are flimsy, yet the general focus on male experiences in Calais no doubt emerges not only from their presence in far greater proportion, but for precisely these ethical and practical challenges of carrying out in-depth research with women in a particularly vulnerable position at this border. Even in quantitative research emerging from Calais, women tend to be underrepresented: a 2015 quantitative survey on health and violence endured by refugees during their journey and in Calais acknowledges that ‘one of the major limitations of this survey is that the proportion of women included was very small. We were unable to conduct the survey successfully in the separate facility housing women and children, so our results cannot be considered representative of women and children refugees’ (Bouhenia et al., 2017: 341).

Carrying out research in northern France since 2016, I have held a lingering sense of complicity in neglecting female border experiences. I engaged with women but only superficially, shying away perhaps from these encounters which often proved more emotionally challenging than encounters with the dominant groups of men. Gökarıksel et al. (2021) emphasise the importance of engaging ‘with discomfort and vulnerable bodies within our feminist praxis’ (ibid: 291), to avoid ‘flattening or erasing’ the struggles of some bodies over others (ibid). The lockdown and fact of living with women in a home for several months enabled me to navigate those discomforts, to engage deeply and protractedly with the women I shared that time with in a form of committed (though improvised) feminist ethnography. The feminist focus on embodiment, relationality, and the everyday lent itself well to this unexpected situation; an intuitive and vital practice before becoming a theoretical lens. As Dyck writes, embodiment as a research approach ‘centres the body […] in the analysis of complex processes as social and geographical worlds are made and experienced’ (2011: 358), placing focus on ‘scales and spaces often overlooked by traditional research methods, those regarded as too banal or private to merit consideration’ (Hiemstra 2017: 330). Indeed, the intimacy of lockdown offered an invaluable new prism through which to interrogate structures of power at this border, and to unearth the voices of these women in what Hiemstra describes as a ‘strategy of the periscope’ (2017). It lay bare cracks in what I had thus far encountered as a predominantly masculine vision and narrative of border space; with women present, but always occupying a peripheral role. In this sense, the lockdown became a moment of refocusing and rediscovery for me, as it was for the women with whom I lived.

As a white, female scholar, British-French citizen and volunteer at the house, I found myself in a position of clear privilege relative to the women I was living with. Living in such proximity with border-crossing women, many close to me in age and planning to take significant risks to end their protracted journeys, only emphasised my privilege and the unfairness of the structures pressing them to put themselves in the dangerous situation of informal passage. My role at the house entailed simply living with its residents: taking part in everyday cooking and cleaning as well as activities such as exercising and playing games. A second volunteer and I were also responsible for running the house through tasks like food shopping, scheduling medical and administrative appointments and, when necessary, mediating relationships between house residents. Despite these roles which establish a form of hierarchy, living together under lockdown required collective house management and decision-making.

Nonetheless, I grew concerned about the ethics of carrying out research in these circumstances, particularly in my multi-layered role of support to these women as volunteer, housemate, friend and researcher especially as, over the course of months of living so intimately together, these ‘roles’ folded into one another and we grew close, sharing what another volunteer described to me as a ‘skin to skin experience.’ My immersion and entanglement with the emotional intensities of the house went beyond what I might have sought out as a researcher under normal circumstances. Indeed, I found myself unable to ‘step outside’ of my research during our cohabitation, both metaphorically and literally. This mode of life was however valuable within a feminist ethnography, and out of ethical concern I discussed my role as a researcher with house residents from the outset. Only those who chose to actively participate in the research did so. The empirical material presented here draws on the experiences of eleven women, while also drawing on insights from my broader research and experiences at this border since 2016. Considering the lockdown circumstances, all interviews were carried out in English with the exception of one interview which was carried out in Persian, with a house resident trusted by those being interviewed acting as interpreter. All quotations have been drawn from interviews or notes from discussions, and are presented here with their consent. To ensure anonymity, I have changed the women’s names and occasional identifying details about them, while staying true to the experiences they faced.

## Relegated to domestic space: Rules, restrictions and reduced opportunities

Hostile living conditions at the border prompted many women to take up offers of shelter from locals and NGOs. Nonetheless, even the lives of women with a bed in shelter centred around the encampments, as the social interactions occurring in these spaces are crucial for negotiating passage. In the safehouse I lived at, it was clear that although women were grateful for a place to stay, they resented being *relegated* to domestic space because of their gender. When I asked Raziah, who was travelling with her sister and had been stuck in Calais for over a year, if she thought she would have crossed sooner had they not been sheltered, she replied:

‘If, if, *if* we were living in the jungle, maybe we would be in the UK now. Yes, I think so. Everybody who was in the jungle went early. Even some women who slept in this house but had a father or a brother or a husband in the jungle have all gone already. Because the men would stay in the jungle, they can *learn* everything there. They can make a deal with the smugglers - or if they don’t have money, like us, they can find people to buy a boat with, they can learn how to buy a boat, how to get a driver [to bring them to the coast], they can learn about which place to go from, about… *everything*. If you live in the jungle, you can hear what is going on. […] Why can’t we go? Because we are in a house. […] If we were in the jungle, we would have gone months ago.’

The home space represented immobility, domesticity and stagnation where the encampments represented freedom, possibility and agency, regardless of the unsafe living conditions.

Displaced people living in the precarious encampments scattered around Calais tend to live in silos: although many of the encampments are near one another and sites of humanitarian assistance are often shared, for the most part they are segregated by nationality or language, with little communication occurring between groups, which each have their own more or less hierarchical social organisation and strategy for passage. One of these groups had a ‘rule’ which prevented women travelling alone from attempting passage with the men. The rule was said to exist on the grounds that women were a cause for distraction and arguments as objects of desire, and because women are thought to require help in attempting passage. Samira explained: ‘The problem is that I don’t only need to be *allowed* to try, I need help to get *up* into the truck with my girl. I cannot run and carry her, so someone needs to give her to me after I go up […] They don’t want more women. They want the chance for *them*. […] They are from our country, but they are not all good men.’

This rule was devastating for single women belonging to this group. As a group who seldom relied on smugglers (due to the high cost of passage and lack of a reliable smuggling network for people of this nationality at that time), passage with male peers would have meant free passage for these women, earned by smuggling themselves into lorries on parking areas over which their community held authority. Effectively, the rule meant men claimed opportunity in border space as their own - a nail in the coffin of border virilisation rolled out by the state.^3^ In claiming their right to mobility and dismissing female peers as a burden and distraction, the men placed restrictions on *women’s* right to just that. This exclusion was painful, especially as gender relations were otherwise fairly pleasant: the men welcomed the women’s presence on their day of rest and prayer, and the women, when sheltered, would occasionally bring them home-cooked food. While this varied between national groups, such a rule and comments referring to women as ‘unadapted’ to the border environment reflect how border-crossing women are often perceived by male counterparts as ‘too wilful,’ as having overstepped their social roles. This emphasises how profoundly migrant women’s access to opportunities for crossing borders is constrained by gendered power relations, themselves exacerbated by border securitisation (Tyszler 2019). The moment of lockdown however dismantled this male gatekeeping: what had been a port city ripe with opportunities to smuggle oneself across the border amidst cargo was dried up of traffic almost overnight (Hagan 2023).

The only remaining option for many people in Calais in spring 2020, when small boat crossings were on a steady rise, was to pay the fee of £3.000 for passage through a smuggler. Unidimensional portrayals of smugglers as exploitative villains have often been criticised as counterproductive and flawed (Tinti and Reitano 2018; Campana and Gelsthorpe 2020; Zhang 2007), and while my own research confirms that displaced people are grateful for smugglers, they should not be too quickly romanticised, especially when it comes to their dealings with women. As though drawing from the same handbook, smugglers would pitch themselves to female clients as ‘good Muslims’ or ‘good Christians,’ also drawing on gender stereotypes to emphasise their respect for their own mothers and sisters. They would use these tropes to try and earn women’s trust, and keep them waiting unreasonable lengths of time for passage, for example justifying giving a woman’s place on a boat to a man on the sugar-coated grounds that the weather conditions were ‘too dangerous for women’, regardless of the self-evident fact that ‘the harsh realities of migration make no sexual distinctions (the sea is gender-indiscriminate in who it drowns)’ (Rose 2021: 327).

Where smugglers’ engagements with men tended to be transactional, women often faced gender-related setbacks: their greater reliance on smugglers due to the social and physical challenges of seeking passage alone opened up new opportunities for women’s exploitation. Even when a woman could afford to pay, there would be nothing to stop a smuggler demanding alternate compensation, namely sexual favours, as a border ‘tariff’ (Khosravi 2010; see also Tyszler 2019, 2021). This happened often in Calais, where many of the few women present were travelling alone or with other women.^4^ While some smugglers would ask directly for sex as payment, others would engage in pseudo-gallantry; it was not uncommon for them to invite a woman out for food or take them shopping before asking them to go with them to their home or hotel.^5^ Men, on the other hand, could offer their labour (assisting a smuggler in arranging passage for others) for several weeks or months to ‘buy’ their passage without sexual exploitation. A young Iranian man who eventually earned his passage in this way told me: ‘He [the smuggler] pretends to be a gentleman, but I have seen him bring women to his home many times - sometimes it even happens in the forest with a sleeping bag.’ The sexual exploitation and blackmail of women by smugglers is an underexplored yet long-standing issue in northern France, also documented in Courau’s (2003) ethnography of the Sangatte camp. Sexual violence emerges as a form of punishment of women transgressing into male spaces and activities, reaffirming ‘a woman’s place in the subordinate category of women’ (Rose 2021: 345).

## A domestic counter-politics: Strategic intersectionality in the home

Despite terrible living conditions at the border, there is some sense of claimed agency among displaced people in this space, who act strategically and with purpose. It is a space of strategizing and *active* waiting rather than one of passivity; a place of opportunity if one can access and harness it in one’s favour (Hagan 2022). In the early days of the lockdown however, slowed border traffic meant that displaced people found themselves reduced to the unbearable state of indefinite waiting, a state so often associated with migration (Hage 2009; Ayuero 2012; Ramsay 2017). In the early days of the lockdown, the women’s hope at fast passage was undercut, between fear of the virus and access to the camps cut off. However, it was in these conditions that the home gradually revealed itself as a rare space of cross-community exchange. As explained earlier, groups of displaced people of different nationalities tend to live in silos. Routine evictions and the sporadic fencing up of sites of encampment by the French authorities reinforce the territoriality of these groups, between whom fights over living sites and points from which to attempt passage occasionally break out. By contrast, and in part by virtue of their exclusion from these spaces and inclusion in domestic space, houses hosting displaced women usually bring many different nationalities together.

While the previous section demonstrated how living apart from the camps is often considered a strategic drawback by women, at a time of pandemic it offered precious silver linings. During this period, the possibility of passage without the help of male counterparts was brought home to the women who increasingly turned to one another for support and connections. Had these women been living in the camps alongside their male peers, they might never have come into contact with one another, or never got beyond the mode of self-preservation through distrust one learns quickly along a migration journey. What follows describes how during enforced retreat from the border encampments, women shared a common emotional space, offering one another mutual recognition. I then describe how, in tandem with this recognition, these wilful women worked to braid strategic intersectional alliances *across* their differences in order to ensure their passage.

### Intimacy and recognition in retreat

Though initially strangers to one another, many of the women I lived with found solace in their common status as strangers in the eyes of the states between which they seek to pass (Amin 2012). They nurtured a common domestic space of shared atmospheres, affects and everyday practices. Similarly to the emotional landscape of the refugee camp described by Brankamp (drawing on Saldanha 2010), that of the safehouse enacts ‘a literal many-bodiedness that intensifies sensibilities through social sharing of emotions and displacement experiences […] pain, pleasure, and other sensibilities in this way become clustered and entangled’ (2021: 3). This is valuable for thinking through the social intensities emerging in the safehouse at a particularly emotionally strenuous time of pandemic; indeed space is ‘tactile’, ‘composed of feelings and sensibilities’ (ibid). Of all the forms of violence border-crossing women face, one of the main afflictions was loneliness; the loneliness of embarking on a long and dangerous journey alone, along which you are often greatly outnumbered by men and must always be on your guard. In the context of a safehouse, where nationalities and languages differ, physical proximity and sharing space are significant. Intimacy is built up through proximity and small practices, experiences of everyday life: cooking, eating and cleaning together, nights slept or spent sleeplessly side by side. To a certain extent, a counter to loneliness emerges in this proximity, in an understated recognition between these women that *they* were the ones who had made it this far; their mutual wilfulness strengthening their belief in the possibility of crossing another border.

In front of the mirror one morning, Raziah tugged at her hair with a brush and said: ‘I hate my hair like this - I changed the colour before I came here to look less like “a refugee”’. Of the women I lived with, many had altered their appearances at their own behest or upon the instruction of smugglers, informed by ‘experiences of body policing and a racial gaze’ (Rahbari 2020: 24–25). Others had suffered violence along the way, or seen their bodies change in ways that affected their self-perception. This was evident in the frequency with which women would show me photographs of their ‘former’ selves, stored on their mobile phones like a reminder of how to rebuild themselves. Though it remained a space of transit, in this home space women began to take care of their bodies and minds impacted by their journeys. This was especially true during lockdown, when they found themselves away from the daily gaze of male peers. Small healing processes began, through small practices working counter to the political violence of borders that compresses geopolitics into ‘the intimacies of everyday life and the innermost recesses of the human body’ (Gregory and Pred 2007: 6). In pairs or small groups of three or four, the women would exchange stories, clothes and creams, home-make hair removal wax from lemon and sugar, rub oil into and style one another’s hair, thread one another’s eyebrows with a technique learned by one of the Iranian women from another woman in a refugee camp in Athens. Some of the women started diets and home-exercising, encouraging one another and pooling advice on how to lose weight. The East African women lamented a change in their complexion due to stress and the cold, for which homemade concoctions would near-daily be thought up. Rahbari writes fittingly of ‘the (inter)personal affective capacity of beauty practices in the form of emotional coping, soothing oneself, or creating joy in others’ (2020: 24; see also Jafari and Maclaran 2014). During these moments, a broad range of topics would be discussed, often with great humour, ranging from the serious issues of female genital mutilation, police or smuggler brutality and stress-related irregularities in menstruation, to family, romance and the future. From these conversations, many different perspectives on womanhood and gender relations emerged: from heated debates on the role of men in domestic space to the issue of wearing hijab or unveiling, and whether or not migration could be considered an explicitly feminist act of self-liberation. Such conversations, facilitated by the safe atmosphere of the shared domestic space of the house, were sporadic but organic and seamlessly binding, the acknowledgment of sharing aspects of someone else’s experience offering some relief. These conversations would occur through another understated act of solidarity: many of the women spoke two languages, allowing language chains to seamlessly form from Sorani or Tigrinya into Arabic then Farsi, English and vice versa.

**Figure 1.**
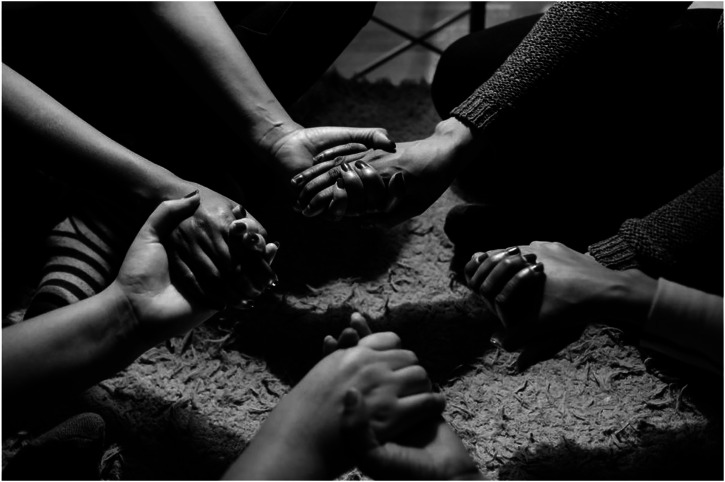
**Alliances in the home** | Photo by Denis Emelin, Calais February 2020.

Particularly close bonds formed between smaller groups, usually according to country or region of origin, but also according to language or emotional need. For example, a mother suffering from distance from her children stepped in to help a young mother in the tiring early months of care for a newborn. This young mother, devastated to have birthed her child far from her own mother, found solace and guidance in this house companion with whom a close mutual bond of friendship and emotional dependency formed. Other women had formed tight-knit groups long before I met them, duos or trios who presented themselves as inseparable, as ‘sisters’ in literal or metaphorical terms (see also Tyszler 2021). These relationships were adopted by some women in an effort to guarantee they would not be separated from one another by humanitarian actors when seeking shelter, nor by smugglers in the moment of passage, which increased their likelihood of being dispersed to the same UK city by the British authorities upon arrival. I do not here wish to create the illusion of perfect harmony; interactions in the house were not always harmonious and mistrust persisted, meaning an awareness of the fragility of this social web was omnipresent. As Hynes writes, mistrust is ‘a logical, useful and rational strategy employed by forcibly displaced people for survival’ (2017: 220); within the house, this was underpinned by the awareness that ‘all women do not share a common social status’ (hooks 2015: 340). However, the particular proximity in which women of different nationalities lived under lockdown somewhat loosened these barriers. Shrieks and prayers of thanks would resound through the house with news of the successful passage of another; moments in which hope is rekindled and everyday differences are cast aside in the reminder of a common goal.

These ethnographic details emphasise how the intimacy imposed on these women under lockdown led to the weaving of emotional connections between them (Figure 1). Bonds formed as a result of proximity and according to need. Whereas women were previously focused on their strategic social relationships with male peers, physical separation from these peers allowed them to turn towards and appear to one another, reaffirming ‘a right that is no right’ (Butler 2011): the legitimacy of *being*, and being *women *at the border. In a context usually so dominated by a framing of women as the less capable, vulnerable ‘others’ (Fiddian-Qasmiyeh 2014), they are encouraged to see one another not only as capable actors, but as women occupying a *legitimate* space. Along a journey during which women are limited by profoundly gendered (and racialised) relations of power and domination, personal and political strength may emerge in the establishment of connections between assembled women who come to recognize a wilful stance in one another, driving them in their own determination to persist (Ahmed 2017; Butler 2011).

### Strategic intersectionality in practice

**Figure 2.**
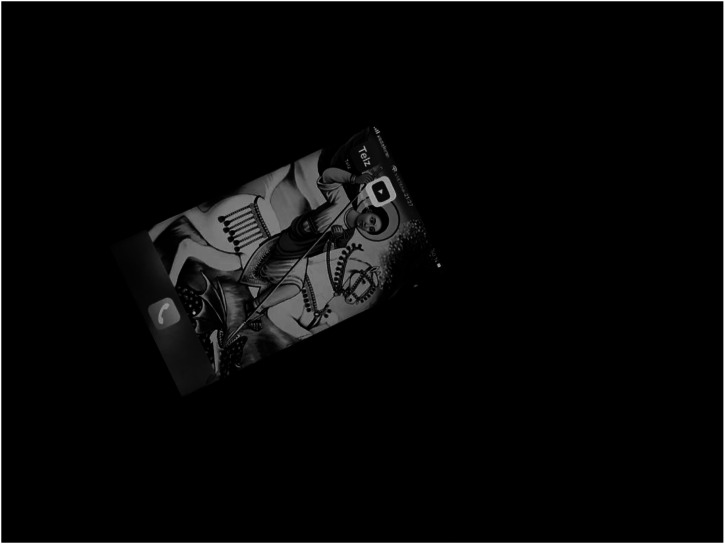
**Waiting for the call** | Photo by author, Calais May 2020.

Following weeks of initial suspicion, the women I lived with came to pool information and ideas to try and navigate smugglers safely and effectively. The common shelter became a site of information exchange, where tactics might be discussed and alliances formed. The home operated as a safe space of which the lockdown made the threshold particularly secure. Indeed, the restrictions were a perfect reason not to meet physically with male peers or smugglers, interactions instead taking place over the phone. Like for the average citizen, much of everyday ‘work’ at the border shifted further online (Figure 2). This unanticipated effect meant that women were better able to protect themselves from violence and consider their options carefully: they were under less pressure to attempt passage immediately than if they had been living outside. Forming what van Liempt describes as a ‘chain of trust’ (2007), phone calls would be made and smuggler reputations triangulated, key phone numbers exchanged or scrawled on scraps of paper and passed from one hand to another in the moment of one’s departure. This was particularly important at a time when, in the absence of cross-border lorry traffic, numbers of people reinventing themselves as boat smugglers was rapidly rising, increasing the risk of being endangered by inexperienced smugglers. For lone women at the time of my research, acquiring a smuggler was not always easy: smugglers were all men, predominantly of one nationality, and hesitant to take passengers of other nationalities.^6^ The connections that emerged in domestic space however helped facilitate exceptions. This empowered the women with the possibility of figuring things out on their own, without needing to pander to men from their respective communities. This emphasises how where the body, domestic space and intimate relationships are so often considered apolitical, these spaces can in fact be of great importance for thinking through the geopolitics of the border, and about how discursive and material relations of geopolitical power may be reproduced and challenged within them (Massaro and Williams 2013).

Sensitive to the value for smugglers of being identified as worthy of referral to peers within a chain of trust (van Liempt 2007), some of the women I met reported strategizing to threaten a smuggler’s reputation in situations where he tried to coerce them into sex for payment, well aware that a good reputation for honesty and trustworthiness is crucial to any smuggler and valuable once earned (Tinti and Reitano 2018; Maher 2018; Campana and Gelsthorpe 2020). As Tinti and Reitano observe, ‘migrants typically have only two main points of leverage with which to bargain with a smuggler. The first, of course, is the amount that they can pay; the second is their capacity to enhance or damage the smuggler’s reputation’ (2018: 46). Seeking to avoid sexual exploitation, one woman instead proposed to help a new smuggler spread his name among peers of her nationality, aware of her strategic intersectional position as a rare communicator *between* national groups. She served as a middleperson and translator to ensure her own passage while opening up a new and invaluable pocket of opportunity for her male peers. Another woman threatened damage to a well-established smuggler’s reputation when he proposed she sleep with him in exchange for passage. Concerned about the damage she could do, the smuggler backtracked and his competitor was quick to step in and offer her a reduced rate, seeing it as an opportunity to boost his own reputation as good-hearted. For women without other options however, the offer of passage in exchange for sexual acts is a serious and traumatic dilemma.

**Figure 3.**
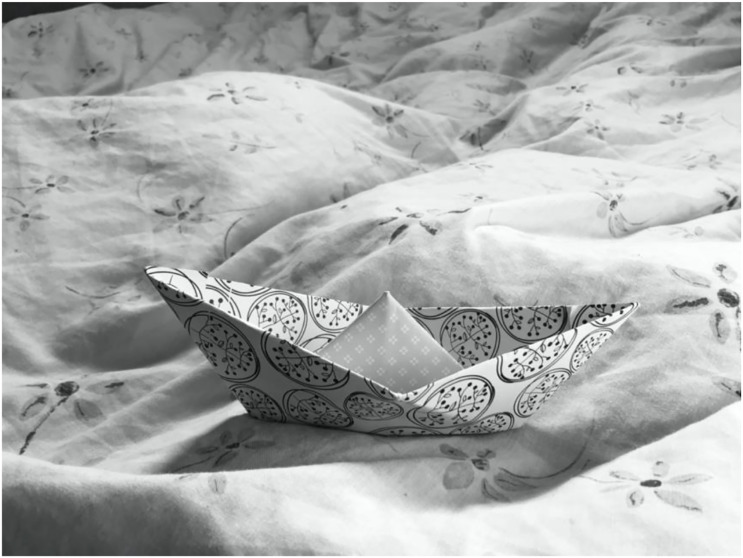
**Making paper boats** | Photo by author, Calais April 2020.

When one woman faced struggles with a predatory Calais smuggler, she told me: ‘you know, I’ve never seen a female smuggler, but I wish I had.’ With a series of difficult crossings behind her and another ahead, she went on to describe how much more trustworthy, safe and just she thought the process would be were the illicit industry run by women.^7^ This struck me as an interesting observation at a time when women (though by no means running their own smuggling operation) were taking as many parameters of their journey into their own hands as possible (Figure 3).

While this article presents a positive outlook on border-crossing women’s wilfulness and its potential when strategically harnessed, this article’s narrative is not a simple one. No matter how airtight a plan elaborated in the home may seem, women, and especially women dependent on men of a different nationality than their own for passage, were particularly afraid of being tricked, scammed or violated after the plan had been set, of sexual violence and of sharing the precarious space of the boat exclusively with men of another nationality during passage - for these boats, too, are male spaces. One woman told me she couldn’t shake the fear that she would be the first to be thrown overboard by male passengers were they to run into trouble on the journey and need to lighten the load. For many women, leaving to cross the border in the dead of the night, with little more than the clothes on their backs, to put their lives in the hands of men in whom they held much hope but no trust, was a fearsome prospect.

## Conclusions

The northern French border, like many other border zones in which informal border-crossing activities take place, is a virilised space experienced as violent by those who seek to pass through it. In Calais, border virilisation emerges from state practices of eviction designed to render border space hostile, which in turn exacerbate the fear and threat of physical violence from male peers that female border-crossers experience. Bearing this in mind, this article has argued that the first pandemic-prompted lockdown worked to disrupt male dominance over border space and associated opportunities for crossing the border. This moment gave rise to strategic intersectionality among displaced women, a form of domestic counter-politics that emerged through the establishment of female coalitions. Women’s wilfulness and resourcefulness coalesced as a discrete domestic form of strategic intersectional resistance, an ‘acting together that opens up time and space outside and against the temporality and established architecture of the regime’ (Butler 2011). This article deviates from dominant discourses about women who migrate as disempowered by taking their own narratives, experiences and strategies into account. Although the body, domestic space and relationships are often overlooked, approaching studies of border life through these sites emphasises how close attention to the intimate may reveal certain border realities and the relations of geopolitical power that are reproduced and challenged within them (Massaro and Williams 2013). Not only do these women circumvent the immobility to which the state endeavours to submit them, but also find ways of circumventing limitations to which the social processes emergent in border geographies would have them submit. Instead of engaging with the virilised and ‘public’ space of the border, formerly considered vital for survival and accessing opportunities for passage, border-crossing women rework domestic space: their mobility is paradoxically facilitated by the stuck conditions under which the lockdown meant they were living.^8^

This article expands on existing work on the Calais border in several ways. It presents a snapshot of the border city at the peculiar time of lockdown, but also goes beyond analysis of this moment. The unanticipated methodology of ‘the periscope’ (Hiemstra 2017), located in the intimate and facilitated by the lockdown, has refracted a vital account of female subjectivities at this border more broadly than just in the moment of pandemic. It emphasises the importance for migration scholars not to overlook female subjectivities even when women are present as small minorities. By emphasising the specificities of female border-crossers’ experiences, the narratives gathered here contribute to broader efforts at fleshing out a feminist geography of borders (Tyszler 2018; Sahraoui 2020), shedding light on how state and social power relations intersect at the intimate scale of the female body. As Davis writes: ‘feminist knowledge production, when linked to methodological strategies, should unravel issues of power and include interventions that help move toward social justice’ (2013: 27). Though often prompted by desperation, the act of migration is profoundly political, especially for women moving alone in pursuit of an actively chosen destination. With this article, I hope to shed light on the uneven gendered consequences of border politics, prompting a long overdue scholarly conversation on border-crossing women and shifting away from predominantly masculinist portrayals of border space.

## Notes

1. All names and some details have been changed throughout this article to protect the anonymity of research participants.

2. The term ‘jungle’ is commonly used by humanitarians and displaced people to refer to the scattered border encampments. The capitalised term ‘Jungle’refers to the makeshift camp that existed at the border in 2015–2016.

3. See Tyszler (2019) for a similar account of the gendered socio-spatial organisation of forest encampments in Morocco.

4. Many women also travel with a partner or as a family, but at the time of research these families (most often Kurdish or Pakistani) tend to live in Grande-Synthe, 30 km from Calais, where they depend on parallel smuggling networks.

5. Some women also mentioned ‘gentlemanly’ smugglers who would make such gestures without expectations, considering it part of their role to support women whose nationality they shared.

6. This departed from a presumption of trust and loyalty from one’s own community, considered less likely to reveal information about the smuggler to British authorities upon arrival.

7. Accounts of women’s involvement in facilitating smuggling are scarce, however there have been noteworthy contributions by Sanchez (2016) and Zhang et al. (2007) regarding women’s participation in smuggling practices between Mexico and the US and China and the United States respectively.

8. Numbers of displaced women in Calais increased in 2020 (Refugee Women’s Centre 2020): over the summer 50–55 women are estimated to have been arriving per month, dropping to 40–45 in the autumn. This rise was no doubt linked to increased opportunities for boat passage. By 2022, the number of women making the crossing was of around 4117 - 9% of the total figure (British Refugee Council 2023). This significant rise in numbers suggests that opportunities for boat passage make this migration route more accessible for women. However, the deadliness of the Channel has also increased, with at least 55 deaths at the border between 2020 and 2022 (of which 8 women and 9 children).
